# A mixed methods exploration of the experiences of physical activity providers in supporting children and adolescents with type 1 diabetes in the UK

**DOI:** 10.1007/s00431-025-06139-z

**Published:** 2025-04-24

**Authors:** Tallulah Ngamy, Laura Statham, Ben Smith, Jane R. Smith, Jenny Lloyd, Ross Clarke, Parth Narendran, Renuka P. Dias, Robert C. Andrews, Emma J. Cockcroft

**Affiliations:** 1 Faculty of Health and Life Sciences, University of Exeter Medical School, Exeter, EX1 2 JP UK; 2 Warwick Medical School, University of Warwick, Coventry, UK; 3 Department of Paediatric Endocrinology and Diabetes, Birmingham Women and Children’s NHS Foundation Trust, Birmingham, B4 6 NH UK; 4 Institute of Applied Health Research, University of Birmingham, Birmingham, B15 2 TT UK; 5 Department of Diabetes, University Hospitals Birmingham NHS Foundation Trust, Birmingham, B4 6 NH UK; 6 NIHR Exeter Biomedical Research Centre (BRC), Exeter, EX1 2 JP UK

**Keywords:** Type 1 diabetes, Exercise, Physical activity, Physical education, Sport

## Abstract

Children and adolescents with type 1 diabetes mellitus (T1D) are less active than recommended and than their peers without T1D. Several barriers to physical activity have been reported, including lack of confidence in the support received from those who facilitate physical activity such as sports coaches and PE teachers (physical activity providers). This study aims to explore the experiences and needs of physical activity providers in supporting children and adolescents with T1D to engage in structured physical activity. A mixed-methods study, including an online survey and semi-structured interviews, was conducted to provide a broad understanding of the current context along with more in-depth exploration of strategies implemented. Thirty-four participants completed the survey and nine participated in semi-structured interviews. Survey data were summarised descriptively. Qualitative data were analysed using a reflective thematic approach. Fewer than one-third of respondents had received T1D-related training, and only 12% were aware of relevant policies at their institution. Despite moderate confidence among half of the respondents in supporting children and adolescents with T1D, nearly all expressed a need for specific training. Two overarching themes emerged from the interviews: the existing context within which providers operate and effective strategies to support T1D-related physical activity.

*Conclusion*: Findings suggest improvements can be made to (1) training for physical activity providers to allow them to better support children and adolescents with T1D to participate in physical activity and (2) policies to help ensure a supportive and safe environment for children and adolescents with T1D.

**Table:** 

**What is Known:** • *Children and adolescents with type 1 diabetes are less physically active than peers, partly due to limited support and knowledge among physical activity providers.*	
**What is New:** • *Most physical activity providers lack formal training on type 1 diabetes and rely on ad hoc communication with families or clinical teams for support.* • *There is a clear need for targeted training and policy development to improve safe and inclusive physical activity for children and adolescents with type 1 diabetes.*

**Supplementary Information:**

The online version contains supplementary material available at 10.1007/s00431-025-06139-z.

## Introduction

Type 1 diabetes (T1D) is one of the most common chronic conditions in children and adolescents, affecting about 1.5 million people worldwide under the age of 20 [1, 2]. Whilst there is no cure, management relies on exogenous insulin, blood sugar monitoring, diet, and physical activity [3]. Physical activity not only helps manage T1D but also offers broader health benefits [4–6], including reducing long-term cardiovascular risks and increasing the likelihood of being active in adulthood [5]. However, up to 60% of children with T1D do not meet recommended physical activity levels and tend to be less active than their peers [7, 8].

Physical activity providers, such as physical education (PE) teachers, sports coaches, and community activity leaders, are critical in promoting participation among children and adolescents. However, these professionals often lack the necessary knowledge to facilitate inclusive participation, limiting opportunities for T1D children and adolescents to engage in physical activity on par with their peers. A systematic review highlighted the importance of support systems, including teachers, in enabling physical activity for this population [9]. Recent focus groups involving young individuals with T1D and their parents have also identified challenges such as stigmatisation, which in some cases leads to withdrawal from team sports, despite their associated mental health benefits. A lack of understanding from teachers and coaches further exacerbates participation barriers, with concerns over inadequate staff knowledge during physical activity sessions [10].

Surveys conducted by various organisations reinforce these concerns. A survey by the Association for Physical Education revealed that many parents are not confident in their child’s teachers’ ability to manage diabetes-related emergencies, whilst children themselves are hesitant to approach staff about T1D-related issues [11]. Similarly, a survey by Breakthrough T1D found that only 6% of respondents believed sports coaches, PE teachers, or gym instructors were knowledgeable about T1D and exercise [12]. In Germany, a survey of 120 PE teachers reported that over half rated their diabetes-related knowledge and first-aid skills as inadequate, with only 21% willing to administer emergency glucagon injections [13].

Although physical activity providers play a pivotal role in supporting children and adolescents with T1D, both parents and children and adolescents express low confidence in the support offered, and providers themselves often report insufficient knowledge and confidence. Research exploring the perspectives of PE teachers, sports coaches, and other physical activity providers remains limited, particularly beyond basic knowledge assessment. This study therefore aims to understand the complexities and strategies needed to better support adolescents with T1D in physical activity from the perspective of PE teachers, sports coaches, and other physical activity providers, specifically those support secondary school-aged children and adolescents (12–18 years).

## Methods

A mixed-methods study, including an online survey and semi-structured interviews, was conducted to provide a broad understanding of the current context along with more in-depth exploration of strategies implemented, and factors that may influence how physical activity providers support children and adolescents with T1D. Clinical trial number: not applicable.

### Data collection

#### Online survey

Physical activity providers took part in an online survey, hosted by Bristol Online surveys (Appendix 1) between October 2022 and July 2023. This was designed as an exploratory study to gain an initial understanding of the topic (www.onlinesurveys.ac.uk). The 22-question survey was developed using a mixture of closed and open questions and consisted of 4 sections: background and demographics, general experiences, training and support, and support needs. Participants were asked to rate statements on a five-point Likert scale from ‘strongly agree’ to ‘strongly disagree’ on a range of themes designed to ascertain their views. Additional questions asked about school/club policies, training provision and perceived value of supporting children and adolescents with T1D. The content of the survey was informed by our previous research with healthcare professionals [14] as well as advice from the project’s young person and parents’ advisory group. Consent was provided by all participants prior to completing the survey, including the option to consent to be contacted for a follow up interview.

All PE teachers and Sports coaches in the UK, with some experience of supporting children and adolescents with T1D, were eligible to take part. Potential participants were recruited through targeted emails to schools, sports clubs, and governing bodies, and supplemented with social media adverts (X and Instagram). Emails were sent to 164 organisations asking them to cascade the survey to members and appropriate people. Although we cannot provide details of how many individuals received information about the study the survey link was accessed 1629 times.

#### Semi-structured interviews

All participants who agreed to be contacted for a follow up interview (*n* = 17) were approached via email. Nine participants consented and the semi-structured interview were conducted between January and July 2023. Interviews took place over video call using either Zoom or Teams at a mutually convenient time. The majority of interviews (*N* = 7) were conducted by EC a female postdoctoral researcher with 8 years’ experience in mixed methods research. Two undergraduate students BS and TN interviewed one provider each (under the guidance of EC) following training in qualitative methods and interview techniques. Interviews were structured using a topic guide (see Appendix 2) which was co-developed with the project’s young person and parents’ advisory group. Interview length ranged from 16 to 47 min.

### Data analysis

#### Online survey

Survey responses were downloaded from the survey software and anonymised. Quantitative responses were analysed descriptively, with frequencies presented for each category of response. Where percentages are included, they are rounded to the nearest %. Survey responses were checked by the first author. No responses excluded from analysis due to missing data.

#### Semi-structured interviews

The audio-recorded interviews were transcribed verbatim and imported into the qualitative software programme NVivo (V14.0). Analysis followed a thematic approach [15] consisting of 5 stages: (1) familiarisation with the transcripts; (2) systematic line by line coding of transcripts, using descriptive codes, that were primarily inductive, and data driven; (3) reviewing the codes across the transcripts and grouping these into broader thematic categories; (4) Development of coding framework to apply across transcripts; (5) discussion and refinement of codes and themes with the wider research team (RD, RA, JL, JS, RC) to develop the final set of codes and themes presented here. Stages 1–3 were undertaken by two researchers (LS and EC); stage 4 involved double coding of all transcripts (LS, EC, and TN).

## Results

### Online survey

#### Sample characteristics

Thirty-four responses were received from physical activity providers (22 PE teachers, 10 sports coaches, and 2 outdoor activity supervisors) who supported adolescents (12–18 years) in physical activity. Descriptive characteristics of survey respondents are shown in Table 1. The respondents included a mix of males (*n* = 19) and females (*n* = 15). Years of experience in roles ranged from 1 to 38 years (median of 7 years). Eight participants had personal experience of T1D, with either themselves or family members having the condition.

**Table 1:** Characteristics of survey participants

	*N* (%)
Sex	
Male	19 (56%)
Female	15 (44%)
Age (years)	
21–30	8 (24%)
31–40	11 (32%)
41–50	9 (26%)
51 +	6 (18%)
Highest level of education	
Secondary education	3 (9%)
University degree	17 (50%)
Postgraduate degree	14 (41%)
Profession	
PE teacher	22 (65%)
Sports coach	10 (29%)
Other	2 (6%)
Duration of experience (years)	
1–5	12 (35%)
6–10	9 (26%)
> 10	13 (38%)

Due to rounding, not all percentage totals equate to 100%

Only four respondents (11%) said they were aware of their school/club having a policy for supporting children and adolescents with T1D. This was substantially lower than for other chronic conditions (*n* = 10, 30%) such as epilepsy, asthma, and allergies.

#### Physical activity provider confidence in supporting physical activity

Physical activity providers’ confidence in supporting aspects of diabetes management around physical activity is shown in Table 2. Only half of respondents (*n* = 17) felt confident or very confident in supporting children and adolescents with T1D to engage in physical activity. Less than half (*n* = 14) were confident with pre-exercise blood glucose checks or with pre-exercise carbohydrate intake, and less than a third (*n* = 10) with pre-exercise insulin adjustment. Only around a quarter (*n* = 9) felt confident or very confident in dealing with a diabetic emergency. Just over half (*n* = 19) of respondents felt confident adapting sessions to be inclusive for children and adolescents with T1D.

**Table 2:** Physical activity providers confidence in supporting physical activity in children and adolescents with T1D

Item	Not confident, *n* (%)	Somewhat confident, *n* (%)	Moderately confident, *n* (%)	Confident, *n* (%)	Very confident, *n* (%)
How confident do you feel in supporting an adolescent with type 1 diabetes to engage in physical activity?	4 (12%)	5 (15%)	8 (24%)	10 (29%)	7 (21%)
How confident do you feel in supporting an adolescent with type 1 diabetes to undertake the self-care actions needed in preparation for physical activity to prevent high or low blood sugar?					
Pre exercise blood sugar check?	5 (15%)	5 (15%)	9 (26%)	5 (15%)	9 (26%)
Pre exercise adjustments to insulin?	11 (32%)	3 (9%)	9 (26%)	4 (12%)	6 (18%)
Pre exercise carbohydrate intake?	6 (18%)	5 (15%)	8 (24%)	6 (18%)	8 (24%)
How confident do you feel in undertaking actions to cope with a diabetic emergency relating to taking part in physical activity?	8 (24%)	4 (12%)	13 (38%)	5 (15%)	4 (12%)

Due to rounding, not all percentage totals equate to 100%

#### Knowledge about T1D and training needs

Two-thirds (*n* = − 23/34) rated their knowledge of T1D as average or below. Of the eleven respondents with good or excellent knowledge, eight participants had personal experience of T1D. Most (*n* = 29/34) felt it would be very important or important to develop training materials for helping physical activity providers to support children and adolescents with T1D to be physically active with almost all (*n* = 32/34) saying they would make use of any training resource.

Thirty respondents had received formal training for their role but only ten had received any sort of training relating to T1D. Formal training relating to the role included degrees and teaching qualifications for teachers, and coaching qualifications for sports coaches (for example ‘FA level 1 Coaching’) Training relating to T1D most commonly as part of routine first aid training (*n* = 3/10) or informally through school nurses (*n* = 3/10), charities (*n* = 1/10), parents (*n* = 1/10), or hospital staff (*n* = 1/10). Training was more common among PE teachers (*n* = 7/22) than sports coaches and two outdoor activity supervisors (*n* = 2/12).

### Semi-structured interviews

#### Sample characteristics

Nine physical activity providers supporting adolescents were interviewed, including four PE teachers and five sports coaches. Participants had been in their respective roles for 5 or less years, 6 to 10 years, or more than 10 years. Characteristics of participants are shown in Table 3. Of the four PE teachers, two were independent schools, two state schools. The Sports coaches were involved in supporting a range of sports with specific experience of supporting rugby, football, netball, volleyball, and rowing. Participants were located across the UK, with participants in Scotland (*n* = 2), Wales (*n* = 2), and six in England (Southwest (*n* = 2), Southeast (*n* = 2), West midlands (*n* = 2)). All but one of the sports coaches interviewed were involved in a voluntary manner.

**Table 3:** Characteristics of interview participants

	**Age (y)**	**Gender**	**Role**	**Years in role**	**Personal or family member experience of T1D**
Participant 1	30–40	Female	Coach	1	No
Participant 2	40–50	Male	PE teacher	21	No
Participant 3	50–60	Male	Coach	6	No
Participant 4	40–50	Male	PE teacher	23	No
Participant 5	40–50	Female	Coach	6	Family member
Participant 6	30–40	Female	PE teacher	3	Family member
Participant 7	40–50	Female	Coach	20	Family member
Participant 8	50–60	Male	Coach	28	Personal
Participant 9	30–40	Male	PE teacher	9	No

Survey responses from interview participants indicated that eight of the nine participants felt confident or very confident in supporting an adolescent with T1D to engage in physical activity. Four of the participants had some direct lived experience of T1D either having the condition themselves (*n* = 1), a partner (*n* = 1) or their child (*n* = 2).

#### Factors influencing physical activity support

**Fig. 1:**
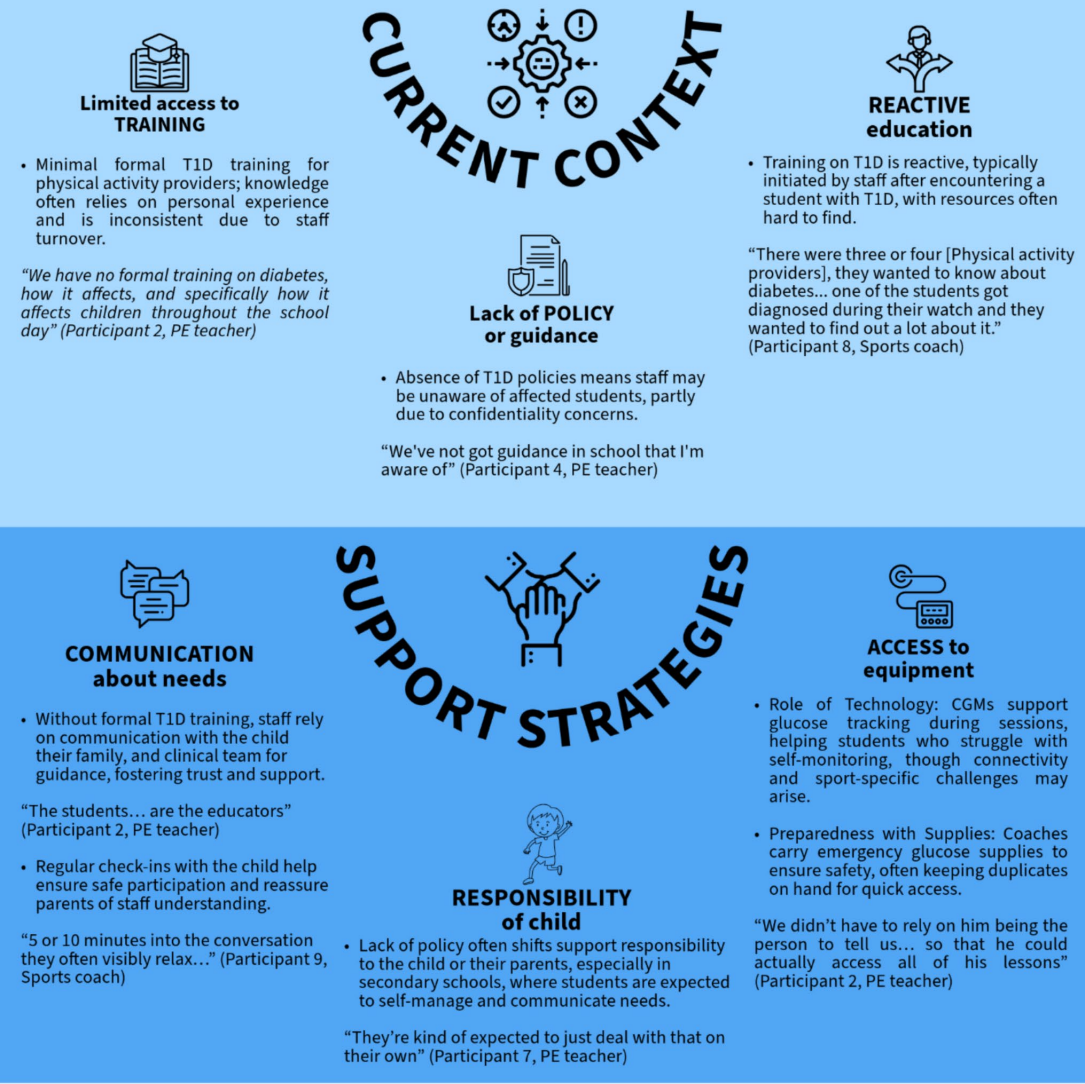
Summary of key categories and themes

Our findings fit into two overarching categories; the current context in which physical activity providers work and strategies for providing support that take account of the current context. Findings from interviews are summarised in Fig. 1. Verbatim quotes are provided to demonstrate themes, labelled with the participant’s role.

#### Current context

Physical activity providers fulfil various roles and therefore require a range of training and practices. Through the interviews, participants shared relevant experiences regarding the current resources, guidelines, and practices available to them or lack thereof when supporting children and adolescents with T1D.

##### Limited access to T1D training and resources

Interview participants indicated that they had received little to no formal training on T1D, particularly in terms of supporting children and adolescents with the condition or understanding how physical activity might impact its management. The little formal training some had received was through first aid qualifications which covered basics of hypoglycaemia. Instead, physical activity providers cited others with personal experience as their source of knowledge/education. Participants suggested that knowledge within clubs and schools is dependent on a member of staff having T1D or being a parent of a child with T1D.

‘*We have no formal training on diabetes, how it affects, and specifically how it affects children throughout the school day’ (Participant 2, PE teacher)*

The physical activity providers interviewed believed this lack of training and experience has a direct impact on their ability to provide sufficient support. This introduces an element of luck as the chances of a child or adolescent with T1D receiving appropriate support is based on their physical activity providers personal experiences and motivation to gain understanding.

‘*The schools know a little bit, but no one knows huge amounts unless you happen to have a teacher in the school whose children got diabetes*’ *(Participant 4, PE teacher)*

##### Lack of policy and guidance

Several interview participants described not being aware of any policies or guidance for managing and supporting children and adolescents with T1D within their school or club. As a result, it was apparent that some participants were not always made aware of children and adolescents in their school or club who had a T1D diagnosis. It was suggested that this may result from fear of breaking confidentiality meaning information was not passed to potentially appropriate members of staff, including passing on information about other medical conditions.

‘*We*’*ve* *not got guidance in school that I’m aware of’* (Participant 4, PE teacher)

‘*The club don’t really have anything in place for that now that I think about it*’ (*Participant 5, Sports coach*)

##### Reactive rather than proactive education

Due to limited time and limited policies for mandated training, it was indicated that education around T1D was reactive to encountering a child with T1D and initiated by the physical activity providers themselves. Physical activity providers described searching for resources after discovering a child or adolescent with T1D was in their club or class. This was not always successful with some describing difficulties finding appropriate resources.

‘*There were three or four [Physical activity providers], they wanted to know about diabetes, they wanted to learn. I think one of the students got diagnosed during their watch and they wanted to find out a lot about it.*’ (*Participant 8, Sports coach*)

##### Coping strategies used to support children and adolescents with T1D

The lack of policy and training available to providers resulted in a range of coping strategies. These approaches fall under three broad themes, communication, responsibility, and practical steps, as outlined below.

###### Communication about needs

Physical activity providers often relied upon the YP’s family and/or diabetes clinical team to gain knowledge and specific guidance for supporting each child. It was felt that communication was essential in building trust with both the child with T1D and their parents. Physical activity providers also emphasised the importance of the child or adolescent with T1D as a source of diabetes education.

‘*The students have always been very, very educated themselves … they are the educators*’ (*Participant 2, PE teacher*)

In addition, physical activity providers described communication as being an important strategy in itself for supporting children and adolescents, checking in during sessions and prompting them to check their blood sugars.

‘*That’s the thing that’s come across to me when I’ve had those conversations with parents. It’s about 5 or 10 min into the conversation they often visibly relax cause they’re like, OK, this person has obviously dealt with this before and has an understanding and isn’t just flippant… and that kind of stuff is good for reassuring a parent*’ (*Participant 9, Sports coach*)

###### Responsibility placed on child

Without clear policy, physical activity providers explained there was no accountability for them to provide support, so the responsibility during activities was often left to the child or parents who would be present to supervise in the case of sports clubs. There was also an expectation for students to communicate their concerns and needs for them to receive adequate support*.* Physical activity providers also raised concerns that a child or adolescent with T1D may not discuss their glucose levels with them due to embarrassment or fears of letting staff down.

‘*When they go on to first year at the age of 12, they’re classed as young adults, so there’s no one there to support them, so there’s no information given, so they’re kind of expected to just deal with that on their own*’ (*Participant 7, PE teacher*)

###### Ensuring access to diabetes equipment and use of technology

Participants described how equipment and technology aided activity sessions. Continuous glucose monitors (CGMs) were utilised throughout sessions and were particularly helpful for students who struggled to keep track of glucose changes and communicate those changes to their physical activity provider. Some participants raised potential problems with CGMs, for example, the technology failing or the inability to communicate blood glucose levels without phone signal. Other participants described how, for certain sports, CGMs may cause barriers to participation for children and adolescents with T1D.

‘*because of the sensor we were able to have his phone on the poolside and every 15 min check it so that we didn’t have to rely on him being the person to tell us because he was rubbish at communicating…so it made it much more accessible in order to be able to look after him so that he could actually access all of his lessons*’ (*Participant 2, PE teacher*)

Other equipment utilised by coaches and teachers for children and adolescents with T1D included various forms of dextrose and glycogen packed in a bag to be carried by the individual or coach throughout the session in case of emergency. One coach referred to having duplicates of this ‘diabetes kit’ (hypo treatment) for staff.

‘*-we carry one of them [glucose shots] in each of the launches’ ‘I try and remember if they’re going out for a head race or something along those lines, that the cox takes some sweets with him*’ (*Participant 1, Sports coach*)

‘*My first question was always where do you keep your sugar supply? And so then I was like, right, it would actually be a good idea for me to have a bag with all this stuff in and for the kids to have a bag. And so we always have a duplicate of that just to make sure*’ (*Participant 9, Sports coach*)

###### Recognising individual differences

Another factor highlighted was that different children and adolescents want and require different support. This individual variability adds a layer of complexity for physical activity providers trying to provide support.

‘*I really struggle supporting young people with Type 1 Diabetes, and it’s very much you get that attitude ‘oh well we have one child with Type 1 so we kind of know what we’re doing’ but actually no you don’t because every child’s different*.’ (*Participant 7, PE teacher*)

Physical activity providers faced the dilemma of whether to take precautions to protect the YP with T1D or treat them the same as any other child. Some physical activity providers were quite clear that they did not adapt the session; however, this was not always the case, with one participant recognising that a YP with T1D wanted to avoid certain movements due to their glucose monitor and insulin pump. Some children and adolescents were described as independent in managing their condition, but for others, their peers had to prompt them to manage their blood glucose or help them to identify when blood glucose levels were falling low. One teacher mentioned their concerns around supporting students with both T1D and communication challenges.

‘*What I’ve gathered from the lads I’ve looked after in the past is they’re different. They’re not all, you know, one case isn’t the same as another case in terms of how they manage it and how they’re comfortable with managing it*’ (*Participant 2, PE teacher*)

## Discussion

The aim of this study was to understand the experiences of physical activity providers in supporting children and adolescents with Type 1 Diabetes (T1D). Physical activity providers play a crucial role in enabling and promoting participation in physical activity among children and adolescents with T1D, yet previous research in this area has largely focused on PE teachers. Our online survey and interviews provide an overview of the current context in which physical activity providers operate, highlighting approaches taken to support young people with T1D and identifying key barriers. A key theme across the interviews and survey responses was limited formal training being available to physical activity providers around aspects of supporting children and adolescents with T1D. Providers described having to rely on colleagues with personal experience, family members of the young person, peers, or clinical teams to gain knowledge and understanding of T1D. Whilst these strategies offer some level of support, they place additional pressure on young people with T1D and their families, leading to inconsistent support and experiences of participation. This aligns with previous research by Lim and colleagues [16], which found that community sports coaches in Western Australia lacked confidence in supporting young athletes with T1D due to insufficient knowledge and training. Additionally, Sadasivan and Cave [17] reported that youth soccer coaches had limited asthma-related training, despite frequently encountering athletes with asthma. These studies suggest that a broader systemic issue exists regarding the lack of structured education for physical activity providers managing chronic health conditions.

In addition to limited training, data from our survey and interviews also highlight limited awareness of policies relating to type 1 diabetes and suggests policies relating to T1D may be less prevalent than other chronic health conditions. Children and adolescents with T1D should have an individual health care plan (IHCP) which is shared with schools and details the needs of that child during the school day. We did not specifically ask about this document in our survey or interviews, but we suggest that some of our findings in terms of limited policies, lack of understanding about diabetes and in some instances limited awareness of when pupils have T1D, that these documents may not be reaching teachers or being utilised in a sports club setting. This warrants further research.

Physical activity providers are responsible for creating an environment where all young people feel safe and supported to participate in sports. This has been emphasised in previous qualitative work [10] where the lack of knowledge of teachers, the general community, and peers is shown to be a challenge. Whilst children and adolescents were physically active at school, parents expressed concerns about inadequate staff knowledge about T1D and poor internal communication. Limited knowledge of PE teachers has also been reported in work from MacMillan and colleagues who found that teachers, diabetes professionals, and parents highlighted limited knowledge, training, and support for teachers about T1D. In line with our findings, they found that most teachers had acquired their diabetes knowledge from knowing/teaching someone with diabetes, with no teachers reporting training in diabetes management during university [18]. This has also been found for other long-term health conditions [19].

Our findings and those of others are important to consider alongside general strategies to support inclusion, diversity, and equality in schools, organisations, and sports. Inclusion, diversity, and equality are fundamental principles in educational and sporting environments, especially when supporting children and adolescents with chronic health conditions, such as T1D. The UK Equality Act 2010 [18] provides a legal framework that protects individuals from discrimination based on disability, ensuring that children and adolescents with T1D have the right to participate fully in physical activities without facing exclusion or unequal treatment. Schools, organisations, and sports clubs have a legal duty to make reasonable adjustments to accommodate the needs of children and adolescents with T1D, which includes ensuring that physical activity providers are adequately trained and supported to manage T1D effectively. Additionally, the statutory guidance Supporting Pupils at School with Medical Conditions [20] outlines the legal responsibilities of schools in ensuring that students with medical conditions, including T1D, are properly supported. This guidance mandates that all schools should have policies in place for managing medical conditions, ensuring that staff are adequately trained and that pupils can access and participate in school activities safely. However, our findings indicate that whilst these policies exist, their implementation varies widely, leading to inconsistencies in the level of support provided across different educational and sporting settings. This discrepancy is particularly evident in the differences between state and independent schools, where resource availability and institutional priorities may influence the extent to which policies are enacted. Relating to sports coaches, the UK Sport Equality, Diversity, and Inclusion Strategy [21] further emphasises the need for inclusive environments that not only comply with legal standards but actively promote equality and celebrate diversity. This strategy calls for sport organisations to adopt inclusive practices, foster a sense of belonging, and ensure equal participation for all individuals, including those with chronic health conditions. In previous research, PE teachers with experience in working with children’s disabilities shared that creating a structured and welcoming environment through communication, inclusivity, and comprehension in addition to adapting teaching styles and sessions were key in facilitating such inclusive practices [22], which aligns with findings from our work focused on children and adolescents with T1D.

### Study limitations

This study does have some limitations. The sample size was small, with only 34 survey respondents and nine interview participants. The survey responses allowed initial insight and a method for recruitment to the in-depth interviews, but should be considered exploratory, with a larger survey needed to gain a widespread understanding of the current situation. Despite efforts to recruit nationally, we did not record location of survey participants so are unable to give a geographic spread of responses. The low uptake of responses may in part highlight the limited awareness of T1D in this population. We were interested in hearing from those who had experience of supporting children and adolescents with T1D, but on reflection, this may have been a barrier to recruitment. In conjunction with this, the participants were likely to be more motivated and knowledgeable about the topic than the wider population. We can therefore speculate that the lack of knowledge on how to support YPs with T1D within the wider population would be greater.

The nine in-depth interviews are in-line with sample sizes from similar previous projects and were considered adequate to answer the research questions and included a spread of participants across the UK. However, it is worth noting that these participants were even more self-selected and are not representative of the wider population, with four of the interview participants having lived experience of T1D, either themselves or through supporting a family member. However, a range of opinions were captured, and our sample highlighted several barriers that add to the existing body of research in this area, as well as several aspects that corroborate findings from previous research. Additionally, the experience of participants enabled us to gain an understanding of current approaches to support.

### Practical implications

The findings of this study have several practical implications for improving the support provided to children and adolescents with T1D by physical activity providers. First and foremost, there is an urgent need for the development and implementation of education and training specifically designed for physical activity providers. These should cover essential aspects of T1D and its management in the context of physical activity and emergency response procedures, ideally embedded alongside more general training around addressing needs of children and adolescents with chronic health conditions. In addition to training, schools and sports clubs should establish and implement policies that ensure a supportive environment for children and adolescents with T1D, embedding within any broader policies for those with chronic health conditions. How this training is implemented and what is included will vary with different physical activity provider roles, with different contexts (schools vs. sports clubs for example) needing different support mechanisms and training requirements. At a sports organisation level, we feel that each sporting body should have a policy that helps to provide guidance around safety and inclusivity. Organisations should make available some brief training and literature for coaches, in line with what WHO recommends.

Finally, participants’ experiences highlight the critical importance of communication and collaboration between physical activity providers, children and adolescents with T1D, and their families. Engaging in regular dialogue and feedback can enhance mutual understanding, build trust, and ensure that the individual support needs of children and adolescents with T1D are effectively addressed. This is a simple approach that both sports coaches and PE teachers can use to create a supportive environment for children and adolescents with T1D.

Future research should aim to gain a wider understanding of policies in schools and clubs to help support those with chronic health conditions, including T1D, through school and club level data collection. Additionally, we should work with children and adolescents with T1D, and physical activity providers to design education and training which supports the needs of children and adolescents and is appropriate for physical activity providers. Working with key stakeholders will help understand how best to embed policies relating to chronic conditions into organisations.

## Conclusion

This study highlights the significant gaps in knowledge and confidence among physical activity providers in supporting children and adolescents with T1D and underscores the urgent need for comprehensive training and policy implementation. By addressing these knowledge gaps, we can improve the support provided to children and adolescents with T1D, promoting their participation in physical activities and enhancing their overall well-being.

## Supplementary Information

Below is the link to the electronic supplementary material.

Supplementary file1 (DOCX 45 KB)

## Data Availability

The datasets generated and/or analysed during the current study are not publicly available but are available from the corresponding author on reasonable request.
