# Supplementary material for: A mixed methods exploration of the experiences of physical activity providers in supporting children and adolescents with type 1 diabetes in the UK
Source: Eur J Pediatr. 2025 Apr 24;184(5):311. doi: 10.1007/s00431-025-06139-z (PMC12021711; doi:10.1007/s00431-025-06139-z)
Supplement: Supplementary file 1 — Supplementary file1 (DOCX 45 KB) [file 431_2025_6139_MOESM1_ESM.docx]

.

**APPENDIX #1: Copy of the online survey, to be completed via Jisc Online surveys.**

**[information and consent questions on first page]**

***Section #1: Background Demographics [8 questions]***

1. **Age (years):**
2. **Gender:**

- Female / Male / Prefer not to say / Prefer to self-describe… (provide textbox).

1. **What is your highest professional-related academic qualification?** (If you’re currently enrolled in education, please indicate the highest qualification you have received.)
2. **What is your role when working with adolescents?**

- PE Teacher / Sports Coach / Activity Supervisor (i.e Scouts, brownies) / Other… (provide textbox for answer).
- If you have more than one role, or past roles that are relevant, please provide some details ( provide textbox for answer)

1. **How many years have you been in this role?**

- Provide textbox for answer.

1. **Please provide details of what the role involves?**

- Provide textbox for answer.

1. **Do you have any specific qualifications/ training relating to this role? Please provide details of qualifications and where it was obtained.**

- Provide textbox for answer .

1. **To what extent have you worked with adolescents with type 1 diabetes?**

- Provide textbox for answer

*End of Section #1.*

***Section #2: General [7 questions]***

1. **How confident do you feel in supporting an adolescent with type 1 diabetes to engage in physical activity?**

- Likert scale: 1 = not confident / 2 = somewhat confident / 3 = moderately confident / 4 = confident / 5 = very confident / 6 = don’t know.
- Comment box to allow explanation.

1. **How confident do you feel in supporting an adolescent with type 1 diabetes to undertake the self-care actions needed in preparation for physical activity to prevent high or low blood sugar?**
   1. **Pre exercise blood sugar check?**
   2. **Pre exercise adjustments to insulin?**
   3. **Pre exercise carbohydrate**

- Likert scale: 1 = not confident / 2 = somewhat confident / 3 = moderately confident / 4 = confident / 5 = very confident / 6 = don’t know.

1. **How confident do you feel in undertaking actions to cope with a diabetic emergency relating to taking part in physical activity?**

- Likert scale: 1 = not confident / 2 = somewhat confident / 3 = moderately confident / 4 = confident / 5 = very confident / 6 = don’t know.

1. **What action might you take if a young person’s blood sugar becomes too low? (**Please give your initial reflection without searching for ‘correct’ response. Data remains anonymous and we are interested in your genuine response)

- Provide textbox for answer.

1. **What action might you take if a young person’s blood sugar becomes too high? (**Please give your initial reflection without searching for ‘correct’ response. Data remains anonymous and we are interested in your genuine response)

- Provide textbox for answer.

1. **To what extent do you agree that providing specific support for adolescents with type 1 diabetes is part of your role as a physical activity provider?**
2. Likert scale: 1 = Strongly disagree / 2 = disagree/ 3 = neither agree nor disagree / 4 = Agree / 5 = strongly agree / 6 = don’t know.

*End of Section #2.*

***Section #3: Training and support [3 questions]***

1. **Does your school/club have a policy for supporting adolescents with type 1 diabetes with specific to physical activity?**

- Yes / No / Don’t know.

*If ‘yes’, could you let us know what is in this policy?*

1. **Does your school/club have a policy for supporting adolescents with other chronic conditions (i.e., asthma, epilepsy)?**

Yes / No / Don’t know.

*If ‘yes’, What conditions does the policy cover?*

1. **Have you been provided with any information, training, and/or support to allow you to support adolescents with type 1 diabetes to undertake physical activity?**

- Yes / No / Don’t know.

*If ‘yes’, who provided this information, training, and/or support and please could you let us know what training you have received?*

1. **Do you feel confident in adapting PE lessons/ training /activities to be inclusive for adolescents with type 1 diabetes?**

- Yes / No / Don’t know.

*If ‘yes’, could you let us know what sort of adaptions you may make?*

*if ‘no’, could you tell us why this is the case?*

*End of Section #3.*

***Section #4: Need and Support [3 questions]***

1. **How would you rate your current knowledge about type 1 diabetes and physical activity?**

- Likert scale: 1 = very poor / 2 = poor / 3 = average / 4 = good / 5 = excellent / 6 = don’t know.

*If ‘good’ or ‘excellent’, what sources of information did you use to gain this knowledge?*

- Provide textbox for answer.

*If ‘poor’ or ‘very poor’, why do you think that this is the case, what could improve your current knowledge?*

- Provide textbox for answer.

*If ‘average’ or ‘don’t know’, skip to next question.*

1. **How important to you think it is to develop training materials to help support physical activity providers to support adolescents with type 1 diabetes to be physically active?**

- Likert scale: 1 = not important / 2 = somewhat important / 3 = moderately important / 4 = important / 5 = very important / 6 = don’t know.

1. **If specific training around supporting adolescents with type 1 diabetes was available, would you make use of this resource?**

- Yes / No / Don’t know.

*If ‘yes’, what factors would be important to include in any training?*

- Provide textbox for answer.

*If ‘no’, why would you not make use of this?*

- Provide textbox for answer.

*If ‘don’t know’, skip to next question.*

*End of Section #5.*

***Section #5: Future Involvement [3 questions]***

1. **Would you be willing to take part in a short interview about this topic at a later date?**

- Yes / No.

1. **Would you like to be informed of the results from this research? Results will also be available on the study website (www.ISPA-T1D.com)**

- Yes / No.

1. ***If “yes” to either of the questions above, please provide your contact name and email address and/or phone number below so we can get in touch***

*End of Survey (including submit button)*

**[Debrief section after survey has been completed]**

**Thank you for participating in this study!**

Thank you for taking part in our research about supporting adolescent with type 1 diabetes with physical activity

The purpose of this research was to understand your perspective on supporting adolescents with type 1 diabetes and how we can best support you to do this.

If you have any questions or you are interested in finding out more about this research, you may contact the researcher by email:

**APPENDIX #2: Copy of interview topic guide**

**Improving Support for Physical Activity in Adolescents with Type 1 Diabetes (ISPA-T1D): Understanding physical activity providers experience of supporting adolescents with Type 1 Diabetes**

**Topic guide**

Research objective: Explore the experiences of physical activity providers in supporting adolescents with type one diabetes be more physically active.

**[Before turning recorder on]**

- Introduce myself
- Go over information sheet and provide details about the focus of the research, explain this is about type 1 diabetes (outline difference between type 1 and type 2)
- State that the participant has previously provided Consent to participate in this study. Ask them to verbally consent to being involved with this interview.
- Explain process and that I will be making notes during the discussion as a reminder to myself to come back to
- Reiterate there’s no right or wrong answer. Interested to know about their experiences. Reiterate that the research is anonymous and is not a review or ‘test’ of your current practice, we are interested in current experiences, encourage to be as open and honest as they can be their responses.
- Reassure participant that everything said during the interview (related to their identity) will be anonymised – they will be identified by a made-up name (pseudonym), instead of their real name. Give the participant an opportunity to provide a pseudonym.
- Ensure that the participant understands that they do not have to answer any questions which they do not feel comfortable with. Explain that they can stop taking part at any time, without having to explain why, and can ask for a break at any time.
- Check whether the participant has any questions that they would like to ask.

**[Turn recorder on]**

**Exploring participants experiences of supporting physical activity**

1. **ok, ----------- so today we are going to be talking about your experience of supporting physical activity for adolescents with type one diabetes. In the survey you responded to you indicated that *[outline the experience participant indicated in the survey*], is that right?**
   1. Could you tell me a little bit more about your role and background?
2. **could you tell me a bit about your experience of supporting adolescents with type 1 diabetes with physical activity?**

- Prompt: how many adolescents with T1D have you worked with, how often, [get an idea of intensity [number of young people, frequency and duration] and the type of activity that the adolescents take part in.
- Prompt: can you describe what is different (if anything) in supporting an adolescent with Type one diabetes compared to adolescents without type 1 diabetes? Do you do anything differently if there is an adolescent with type One diabetes in the group? what specific support is provided to adolescents with type one diabetes

1. **what information, training or support (if any) have you been offered to help you support adolescents with type one diabetes?**

- Prompt: was this training part of your role or did you decide to do this on your own? Who provided the training/support? Did you find it useful?

**Exploring barriers and facilitators to physical activity**

1. **Is there anything you find particularly challenging about supporting an adolescent with type one diabetes in your lessons / club/ training sessions?**

- Prompt: Is it harder in some situations than others, for some people rather than others. Ask to give specific examples.
- Fear of hypo’s, not knowing what to do with insulin, nutrition, knowledge of how diabetes affected by physical activity.

1. **Is there anything you have done/do that you find helpful for supporting and including adolescents with type 1 diabetes?**

- Prompt: can you give any examples of things you do that are inclusive and help enable adolescents with type 1 diabetes.

1. **How important do you think it is for physical activity providers to be aware of and provide specific support for adolescents with type 1 diabetes?**

- Prompt: please explain why you think it is/ isn’t important.
- Where might you go to find information about how to provide specific support.

**Exploring intervention suggestions**

1. **Is there anything in particular you would find useful to help you support adolescents with type 1 diabetes with physical activity and/or joining in with activities [use appropriate language depending on role]?**

- Prompt in terms of capability, opportunity, and motivation.
- Prompt for any parallels/examples/lessons to be learnt in terms of things that help support children with other long-term conditions (e.g asthma)

1. **How would you like to be provided with information to help you support adolescents with type 1 diabetes to be physically active?**

- Prompts: online, face-face, group. Any specific groups or sites that would be trusted.

**Closing**

- *Look through notes to check if anything needs following up*

**1. I’ve asked everything I need to, is there anything else you would like to tell me about?**

**2. Do you have any questions for me?**

**3. [ if verbal consent process has been used then ask again if participant consents for discussion during interview to be used in the research]**

**[Turn off recorder]**

- Thank participant
- Next steps

*Please note that not all follow-up questions are listed in this topic guide. A number of additional questions may be asked based on the participant’s responses. Where possible, these will be open-ended questions.*
